# Diagnostic Pitfalls of Hepatic Sclerosed Hemangiomas: A Case Report

**DOI:** 10.7759/cureus.80738

**Published:** 2025-03-17

**Authors:** Hiroshi Takihara, Shoji Oura, Hiroshi Shintani, Hiroto Tanaka

**Affiliations:** 1 Department of Gastroenterology, Uji Tokushukai Hospital, Uji, JPN; 2 Department of Gastroenterology, Kishiwada Tokushukai Hospital, Kishiwada, JPN; 3 Department of Surgery, Kishiwada Tokushukai Hospital, Kishiwada, JPN

**Keywords:** hepatic sclerosed hematoma, pet, sclerosing cavernous hemangioma, subacute bleeding, tumor marker elevation

## Abstract

An 81-year-old man was found to have a liver mass on an annual medical checkup. Enhanced CT of the mass, 3.8 cm in size, showed weak enhancement with a small non-enhanced oval area near the mass borders. Ultrasound showed an oval mass with internal iso-echoes. Magnetic resonance imaging (MRI) showed that the mass had low and slightly high signal intensities on T1- and T2-weighted images, respectively. MRI of the small non-enhanced area on CT showed high signal intensity both on T1- and T2-weighted images, suggesting focal subacute bleeding. In addition to these image findings, elevated serum α-fetoprotein (AFP) and lectin-reactive fraction of AFP levels made us resect the liver mass without performing a biopsy to the tumor under the tentative diagnosis of possible hepatic malignancy. A postoperative pathological study showed that the mass had massive scar tissue with hemorrhage, lymphocytes, hemosiderin-laden macrophages, and multiple vascular structures, leading to the diagnosis of a hepatic sclerosed hemangioma (HSH). Why this case showed high tumor marker levels remains uncertain. The patient showed normal tumor marker levels shortly after surgery and has been well for 40 months without any problems. Diagnostic physicians should note that HSHs can present very similar image findings to those of intra-hepatic cholangiocarcinomas.

## Introduction

Hepatic hemangiomas are the most frequent benign liver tumors and can be easily diagnosed with various image modalities due to their characteristic blood flow and pathological structures [1]. Hepatic sclerosed hemangiomas (HSHs) and sclerosing cavernous hemangiomas (SCHs) are rare subtypes of hepatic hemangiomas, both with degenerative fibrosis. Jia et al. distinguished between HSHs and SCHs based on the degree of denatured changes [2]. Although the former degeneration is defined as more severe than the latter, no clear differential diagnosis between the two disorders has been established to date. HSHs and SCHs can show various imaging findings very similar to those of hepatocellular carcinomas (HCCs), intra-hepatic cholangiocellular carcinomas (iCCCs), and metastatic liver tumors. In addition, some SCHs can have elevated serum α-fetoprotein (AFP) levels [2].

Regarding the differential diagnosis of HSHs and HCSs, the involvement of mast cells in angiogenesis and differences in immunoreactivity for various molecules such as collagen IV and smooth muscle actin have been discussed [3]. However, confusion exists regarding the definition and nomenclature of HSHs and SCHs, possibly due to the paucity of their cases. Many authors, therefore, have grouped these disorders together as hepatic sclerosing hemangiomas [3-7]. Although the exact definition of these disorders remains unclear, both SCHs and HSHs have similar pathological findings that narrowing and obstruction due to fibrosis and hyalinization are observed in the hepatic hemangioma lumens.

We herein report a case of an HSH with elevated serum tumor marker levels and similar image findings to those of iCCCs.

## Case presentation

**Figure 1:**
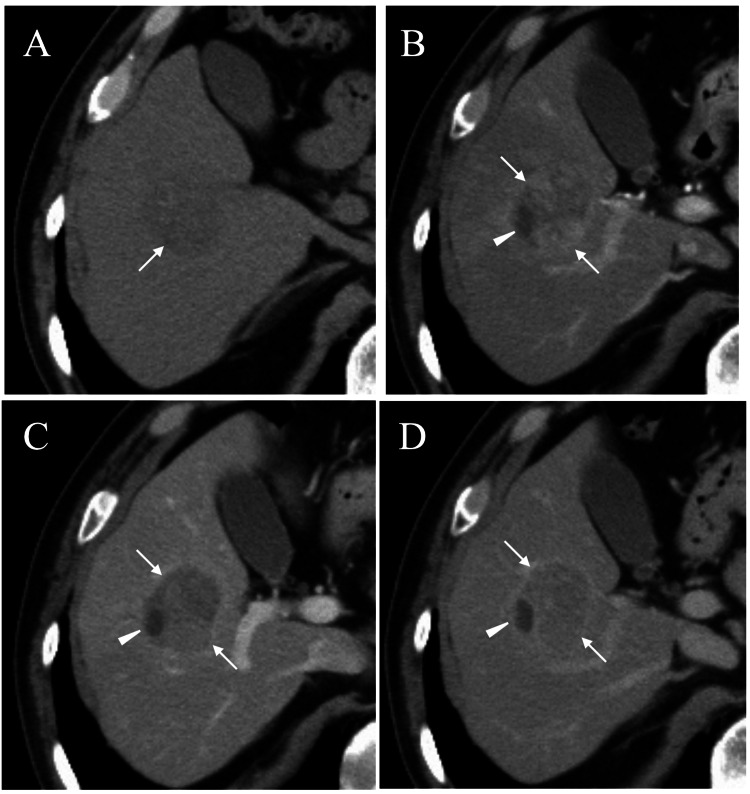
Computed tomography (CT) findings A. CT showed an oval mass (arrow) with a slightly lower CT Hounsfield Unit value compared to that of the surrounding liver parenchyma. B. CT showed a weak enhancement predominantly in the mass margins (arrows) and a small part with no enhancement (arrowhead) on the early arterial phase. C. CT showed a washout pattern (arrows) and a clearly demarcated non-enhancement small area (arrowhead) on the late arterial phase. D. CT showed a retained slight rim enhancement (arrows) and a non-enhancement area (arrowhead) on the portal phase.

An 81-year-old man was incidentally identified with a mass in the liver on a periodical follow-up for a mass screening, which detected a lung lesion. Computed tomography (CT) showed an oval mass with a slightly lower CT Hounsfield Unit (HU) value than that of the adjacent liver parenchyma (Figure 1A). CT of the mass showed weak enhancement on the early arterial phase (Figure 1B) and a washout pattern on the late arterial phase (Figure 1C).

**Figure 2:**
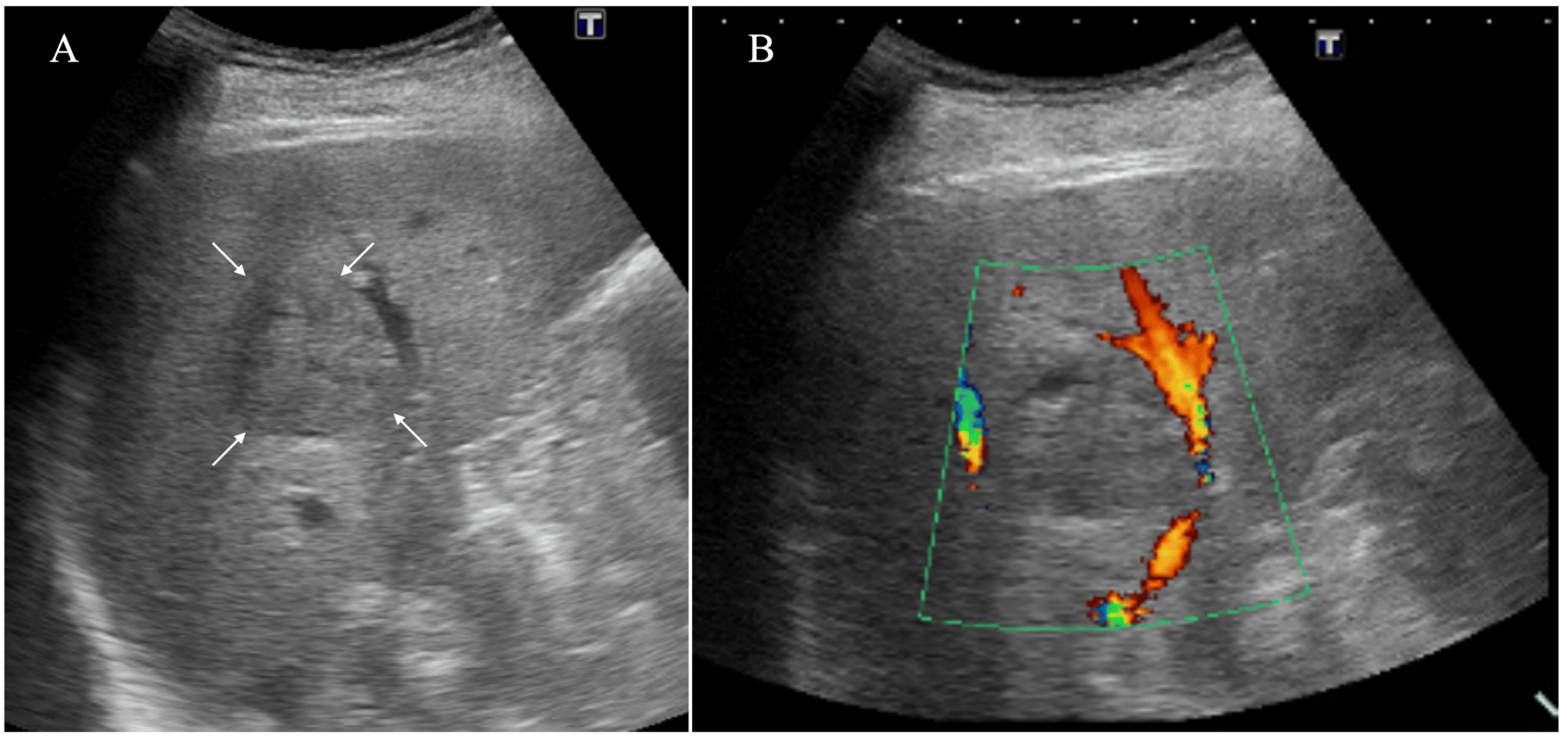
Ultrasound findings Ultrasound showed a round mass with enhanced posterior echoes (A, arrows), blood flow around the mass, and no blood inflow to the mass (B).

However, a small area near the mass borders showed no enhancement up to the delayed phase (Figures 1B-1D). Ultrasound (US) showed an oval mass, 38 mm in size, with internal iso-echoes in the right posterior inferior segment (Figure 2).

**Figure 3:**
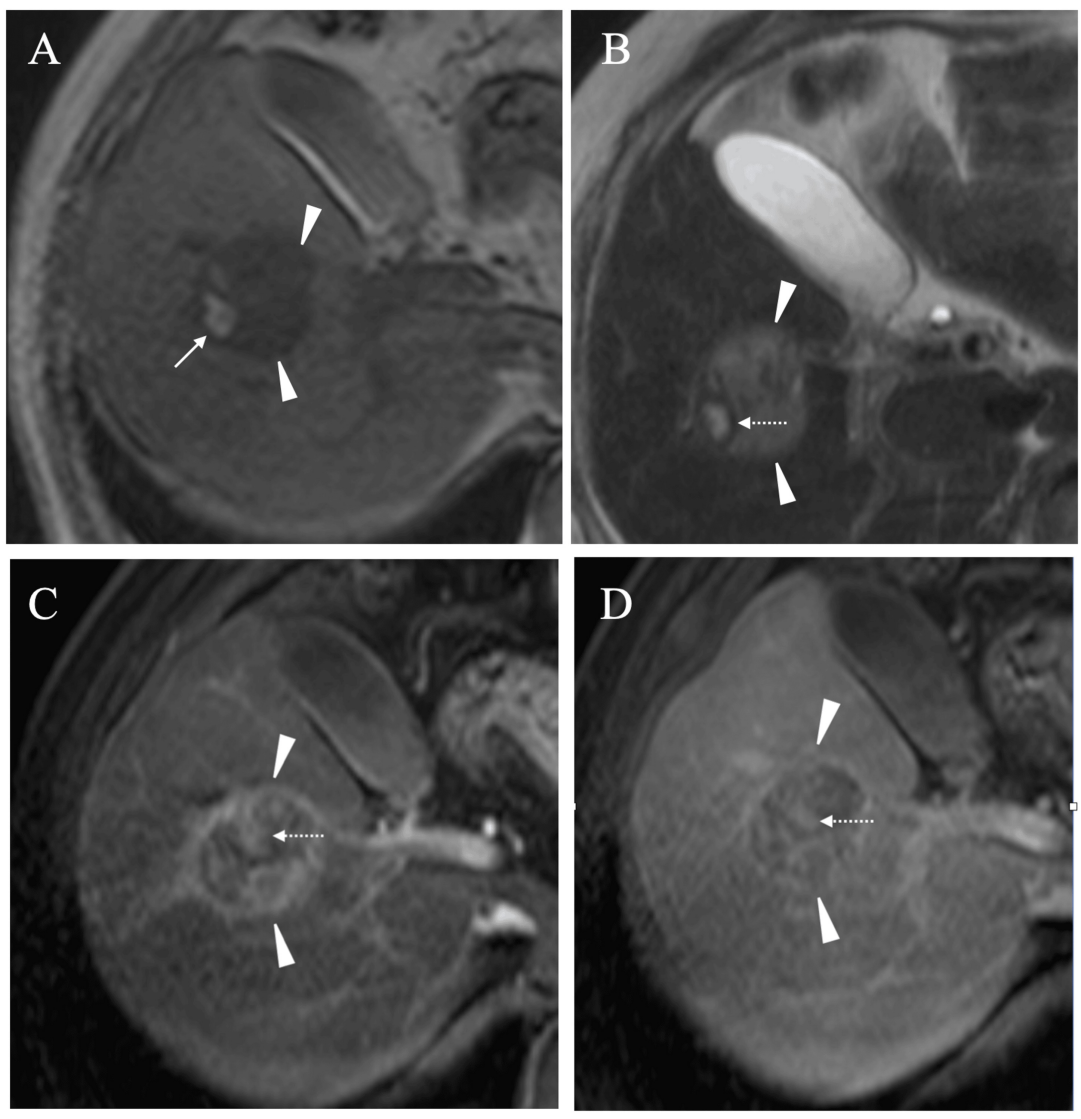
Magnetic resonance image (MRI) findings A. T1-weighted images showed that the mass showed low signal intensity (arrowheads) and had a small high-signal intensity area (arrow) in the mass borders. B. T2-weighted images showed that the mass showed slightly high signal intensity (arrowheads) and had a small high-signal intensity area encircled by a very low signal intensity ring (dashed arrow). C. T1-weighted images 31 seconds after the contrast medium injection showed a weak rim enhancement (arrowheads) and contrast effect directing toward the inside of the tumor (dashed arrow). D. T1-weighted images 120 seconds after the contrast medium injection still showed a weak rim enhancement (arrowheads) and a slight intra-tumoral enhancement (dashed arrow).

Magnetic resonance imaging (MRI) showed that the mass showed predominantly low signal intensity on T1-weighted images and slightly high signal intensity on T2-weighted images. MRI further showed that the small area had high signal intensity both on T1- and T2-weighted images (Figures 3A, 3B), highly suggesting the presence of extra-cellular methemoglobin in that area. Subtraction MRI showed that the mass had weak and slow enhancement (Figure 3C) and retained enhancement even at 120 seconds (Figure 3D).

**Figure 4:**
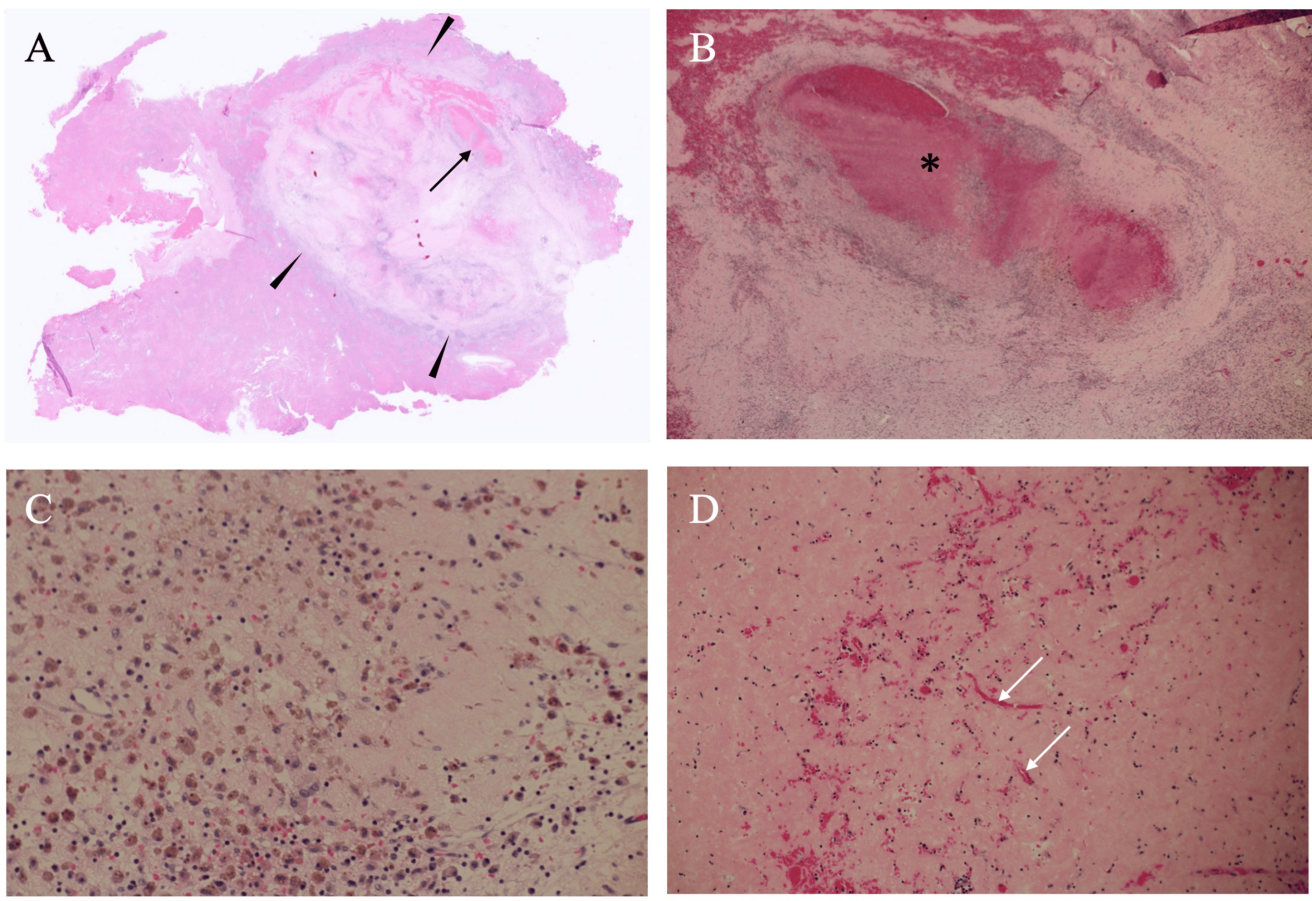
Pathological findings A. Low-magnification view showed an oval scar tissue (arrowheads) and bleeding (arrow) in the scar. B. Magnified view showed bleeding (asterisk) in the scar. C. Magnified view showed multiple hemosiderin-laden macrophages (brownish cells) and lymphocytes. D. Small vessels (arrows) were observed in the scar tissue.

In addition to these image findings, an elevated serum AFP level of 670 ng/mL (reference range: 0-7 ng/mL) and a lectin-reactive fraction of AFP (L3) level of 16.6% (reference range: 0-10%) made us, without performing biopsy of the mass, laparoscopically resect the liver mass under the presumed diagnosis of hepatic malignancy. A postoperative pathological study, however, showed an oval mass composed of abundant scar tissue with hemorrhage (Figures 4A, 4B), lymphocytes, hemosiderin-laden macrophages (Figures 4B, 4C), and multiple vascular structures (Figure 4D), leading to the diagnosis of an HSH.

Although normal AFP and L3 levels were confirmed shortly after the operation, immunostaining did not show any AFP positivity on all the pathological components in the HSH tissue. The patient has been well without any problems for 40 months.

## Discussion

All image modalities in this case showed that the hepatic mass had an oval shape with clear margins. Of the three major hepatic malignancies mentioned above, HCCs and metastatic liver tumors generally have a round or an oval shape with well-defined tumor margins. In contrast, iCCCs often have indistinct tumor margins due to the massive presence of fibrous components at the mass borders but sometimes have clear borders. In other words, many liver tumors, regardless of whether they are benign or malignant, present round or oval mass images with clear margins. It, therefore, is generally difficult to make an image diagnosis based only on the shape and margin clarity of the liver masses.

Most solid tumors show fast and strong enhancement followed by a washout pattern on various images when having high tumor cellularity. Liver tumors, however, can present a different tumor enhancement pattern due to their dual blood supply from the hepatic artery and portal vein. In short, well-differentiated HCCs generally show slow or medium tumor enhancement in the initial phase due to portal vein dominant blood flow. Well-differentiated HCCs, however, generally show strong tumor enhancement at the subacute/delayed phase, while this case showed only weak enhancement up to the late phase, highly negating the possible well-differentiated HCCs. Moderately or poorly differentiated HCCs generally show fast and stronger enhancement, which did not match the imaging findings in this case. iCCCs can have tumor staining patterns similar to those of this case. On the other hand, iCCCs generally show blood inflow to the tumor on US, which was not observed in this case. Diagnostic physicians, however, cannot conclude that, even if no blood inflow to the tumor is observed on US, the tumor is not an iCCC. Therefore, it is difficult to rule out iCCCs based on mass enhancement and blood inflow patterns.

CT only suggested low tumor cellularity and showed a small area without any enhancement. MRI showed the presence of extracellular methemoglobin [8] in the small area and further suggested the presence of fibrosis or hyalinization in the tumor. US showed enhanced posterior echoes of the tumor and therefore suggested the presence of massive hyalinization rather than abundant fibrosis [9].

It is well known that both AFP and L3 have high sensitivity for HCCs. Tumor markers, however, can increase for various reasons even in the absence of tumors. Both AFP and L3 can increase especially when a certain amount of liver cells is damaged. Jia et al. reported that some SCHs had increased tumor marker levels [2]. We, however, think that HSHs also can have elevated tumor marker levels due to the similar mechanisms of tumor marker increase in SCHs.

It seemed difficult for us to distinguish between HSHs and iCCCs based on the conventional image evaluation in this patient. It is well known that small HCCs are well-differentiated ones at high rates and that well-differentiated HCCs often do not show FDG accumulation in tumors on positron emission tomography (PET) [2] due to the retained phosphatase activities in cancer cells [10]. Diagnostic physicians, therefore, often do not apply PET evaluation to small liver masses for differential diagnosis. In fact, we unfortunately did not evaluate this tumor with PET. However, Miyamoto et al. reported in their literature review that there were no cases of SHSs in which FDG accumulation was observed on PET [11]. Although there is a slight possibility of underestimation, the usefulness of needle biopsy in diagnosing liver masses is undisputed. Therefore, if PET and needle biopsy had been performed on this patient, a possible diagnosis of HSH would have been made, which would have resulted in careful imaging follow-up of the mass without surgery.

## Conclusions

Image modalities such as ultrasound, CT, and MRI cannot distinguish HSHs from iCCCs. However, when diagnosing liver tumors suspected of iCCCs, it seems possible to make accurate imaging diagnosis by adding PET evaluation to the conventional image evaluation. Furthermore, it seems essential for diagnostic physicians to keep in mind that in addition to common hemangiomas, there are rare subtypes such as SCHs and HSHs when making the differential diagnosis of liver tumors suspected to be iCCCs.
